# Supplementary material for: Network remodeling induced by transcranial brain stimulation: A computational model of tDCS-triggered cell assembly formation
Source: Netw Neurosci. 2019 Sep 1;3(4):924–43. doi: 10.1162/netn_a_00097 (PMC6777963; doi:10.1162/netn_a_00097)
Supplement: Supplementary file 1 [file netn-03-924-s001.pdf]

Lu, H., Gallinaro, J.V., & Rotter, S. (2019). Supporting information for "Network remodeling induced by transcranial brain stimulation: A computational model of tDCS-triggered cell assembly formation." *Network Neuroscience* , 3(4), 924-943.
https://doi.org/10.1162/netn_a_00097

Supplementary Materials

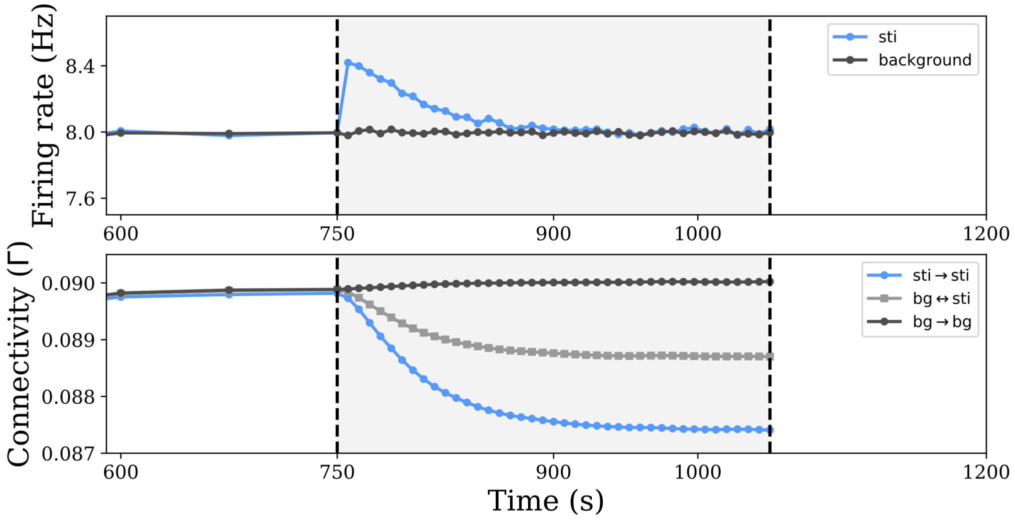

Figure S1: Changes in firing rate and connectivity over time, when a subgroup (10% of all excitatory neurons) is experiencing 2.5 pA DC stimulation. When the firing rate of the stimulated neurons reaches the set-point, the connectivity does not change any more, even if the stimulation is continued. The networks has then reached its structural equilibrium.

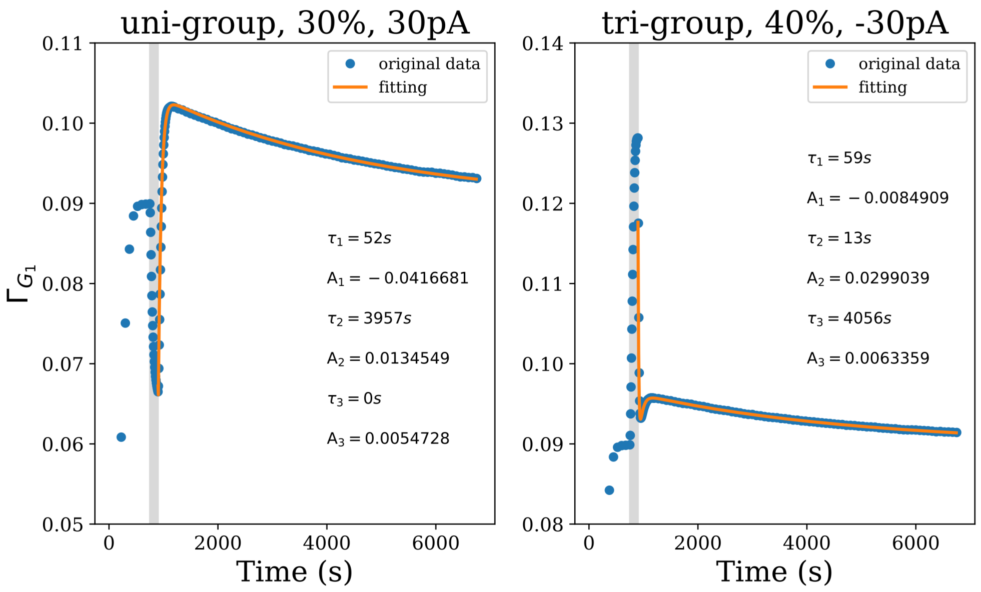

Figure S2: Examples of fitting a triple-exponential function. Left: Fitting the connectivity in the uni-group scenario, where τ_3 amounts to 0 s. Right: Fitting the connectivity in the tri-group scenario. In both cases, the grey bar represents the stimulation phase; the time before the grey bar is the growth phase and after it comes the relaxation phase.
